# Colonization with Carbapenem-Resistant *Enterobacteriaceae* Contributes to Unfavorable Outcomes in End-Stage Liver Disease Patients

**DOI:** 10.3390/antibiotics11111667

**Published:** 2022-11-20

**Authors:** Guofen Zeng, Yihua Pang, Jiaxin Zheng, Chuyue Zhuo, Yingyi Guo, Jiayin Liang, Xiaojie Li, Ziying Lei, Jianyun Zhu, Lejia Xu, Zhiliang Gao, Chao Zhuo, Jing Liu

**Affiliations:** 1 Department of Infectious Diseases, The Third Affiliated Hospital of Sun Yat-sen University, Guangzhou 510630, China; 2 Department of Infectious Diseases, The Affiliated Kashi Hospital, Sun Yat-sen University, Kashi 844000, China; 3 Guangzhou Institute of Respiratory Health, First Affiliated Hospital of Guangzhou Medical University, Guangzhou 510030, China; 4 Department of Laboratory Medicine, The Third Affiliated Hospital of Sun Yat-sen University, Guangzhou 510630, China; 5 Department of Pharmacy, The Third Affiliated Hospital of Sun Yat-sen University, Guangzhou 510630, China

**Keywords:** carbapenem-resistant *Enterobacteriaceae*, colonization, end-stage liver disease, artificial liver support

## Abstract

Carbapenem-resistant Enterobacteriaceae (CRE) are the highest priority pathogens of the World Health Organization, and their prevalence in end-stage liver disease (ESLD) patients is increasing. CRE colonization is an independent risk factor for CRE infections. We aimed to assess risk factors and explore the relationship between CRE colonization, infection, and prognosis in patients with ESLD. A total of 311 patients with ESLD were screened for CRE colonization by fecal swabs from October 2020 to January 2022. Antimicrobial susceptibility was tested using the broth microdilution method. Carbapenem resistance genes, multilocus sequence type, and capsular serotype were analyzed by polymerase chain reaction (PCR). Seventeen CRE strains were detected, among which the most common was *Klebsiella pneumoniae*. The CRE colonization rate was 5.5%. Artificial liver support was an independent risk factor for CRE colonization. Compared to the non-CRE colonization group, the colonization group had a higher incidence of CRE infection and a worse prognosis. Furthermore, these strains were not closely related, and all were sensitive to polymyxin and tigecycline. There was a high colonization rate in ESLD patients, and colonization strains were highly diverse. CRE colonization deserves attention in these patients, especially when treated with artificial liver support.

## 1. Introduction

End-stage liver disease (ESLD) is a severe and life-threatening syndrome, characterized by liver insufficiency, impaired immune function, gut microbiome dysfunction, and barrier impairment [1,2]. There is a bidirectional interaction, called the gut–liver axis, between the liver and the gut with its microbiome. This axis carries bile and antibodies to the intestine and gut products to the liver [1]. It has been proven that cirrhosis has a higher potentially pathogenic microbiome, especially *Enterobacteriaceae* [3,4]. Barrier damage could then result in the translocation of bacteria and subsequent infection [1]. Infections, both a common trigger and a severe complication, often have a devastating effect on the outcome of ESLD, and the emergence of multidrug-resistant (MDR) infections will further increase mortality [3,4,5,6]. Moreover, ESLD patients are often susceptible to MDR infection, which requires frequent hospitalizations, antimicrobial treatment, and invasive operations [7]. It has been reported that liver cirrhosis is a critical predictor of MDR infections in hospital-acquired pneumonia [8]. Patients with cirrhosis were more likely to acquire Carbapenem-resistant *Klebsiella pneumoniae* (CRKP) bloodstream infections than those without cirrhosis [9]. 

Carbapenem-resistant Enterobacteriaceae (CRE), the highest priority pathogens of the World Health Organization (WHO) due to their high morbidity and mortality, cause the most common MDR infection in ESLD [5,10]. CRE infection incidence is rising rapidly globally [11,12], and the prevalence of MDR infections in ESLD is increasing [6,13]. Colonization is a prerequisite and an independent factor for CRE infection [14,15]. Furthermore, colonization could be an important source of transmission. Active screening for CRE colonization will help control its spread [16,17,18]. A German study showed that colonization with MDR bacteria increases mortality in patients with ESLD [19]. Since ESLD has a predilection for *Enterobacteriaceae* and risk factors of MDR, it raises the question of whether ESLD shows disease preference in CRE colonization. In this study, we assessed the prevalence of CRE colonization in patients with ESLD and analyzed the microbiological characteristics of the isolates and their potential transmission routes. Furthermore, we identified the risk factors for CRE colonization and explored the relationship between CRE colonization, infection, and prognosis in ESLD.

## 2. Results

### 2.1. Clinical Characteristics of the Patients

A total of 311 ESLD patients were enrolled in the study, with a median age of 51.08 ± 12.101 years. Seventeen patients tested positive, and the CRE colonization rate was 5.5% (Table 1). Of these patients, 255(81.99%) were male, the Model for End-Stage Liver Disease (MELD) score was 22.00 (13.45–32.31), and the Child–Pugh score was 9.00 (8.00–11.00). A total of 205 (65.92%) patients had cirrhosis, and 256 (82.32%) had either hepatitis B or C. There were no significant differences between the groups with and without CRE colonization in terms of the Charlson comorbidity index, diabetes, tumor presence, intensive care unit (ICU) admission, gastrointestinal bleeding, blood transfusion, parenteral nutrition, and immunosuppressive or antitumor therapy. There were no statistically significant differences in most laboratory results between the two groups. Notably, the proportion of invasive procedures and antimicrobial therapy before screening was higher in the CRE colonization group, especially in terms of the treatment of artificial liver, than that in the non-CRE colonization group. The incidence of CRE infection was higher in the CRE colonization group. The prognosis of the CRE colonization group was significantly worse than that of the non-CRE colonization group.

### 2.2. Risk Factors of CRE Colonization in ESLD

In the univariate logistic regression analysis, length of hospital stay, invasive procedures, and artificial liver support were risk factors for CRE colonization. Factors such as MELD score, Child–Pugh score, cirrhosis, liver disease causes, diabetes mellitus, malignancy, ICU admission, carbapenem use, quinolone use, and laboratory results were not related to CRE colonization. However, only an artificial liver (95% CI:1.427–11.618; *p* = 0.009) was an independent risk factor (Table 2). CRE colonization was considered a risk factor for CRE infection (95% CI:1.095–306.34; *p* = 0.043) and an indicator of poor prognosis (95% CI:1.038–7.507; *p* = 0.042).

### 2.3. Timeline Analysis

**Figure 1:**
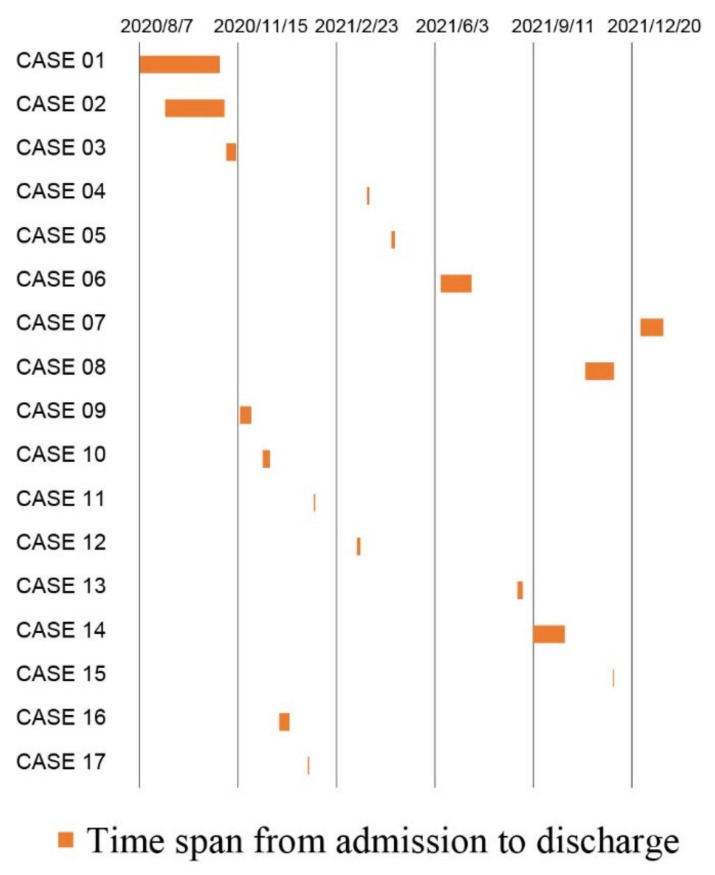
Distribution of length of stay for all the ESLD patients with CRE colonization in this study.

The median time from admission to CRE detection was 7 (2–13) days, with no significant difference between the colonization and non-colonization groups (Table 1). The first detection was made on 27 October 2020, and the last was made on 26 January 2021. The length of hospital stay for patients with strains 1 and 2 overlapped, and there was no overlap in the length of stay for other positive patients (Figure 1).

### 2.4. Antimicrobial Susceptibility Test

**Figure 2:**
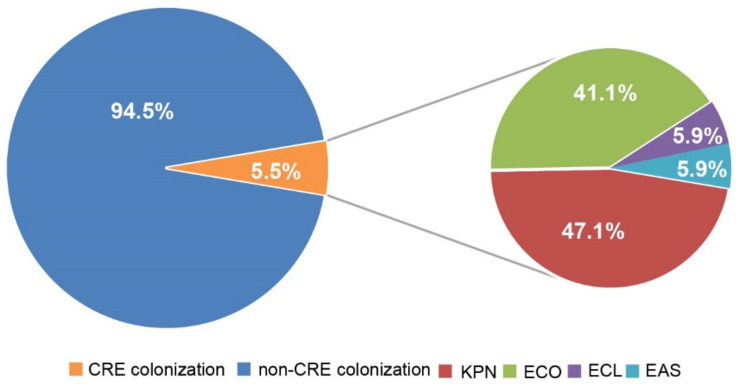
Distribution of the seventeen strains of CRE in ESLD patients.

**Figure 3:**
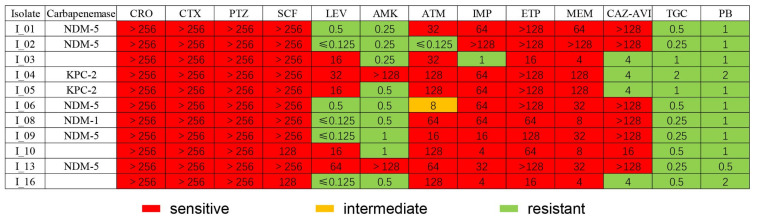
Carbapenemmase and antimicrobial MICs analysis of eleven strains of CRE in patients with ESLD. Isolate I_01/02/03/04/05/06/08/09, Klebsiella pneumoniae; I_10/13, Escherichia coli; I_16, Enterobacter cloacae. CRO: ceftriaxone; CTX: cefotaxime; PTZ: piperacillin tazobactam; SCF: cefoperazone sulbactam; LEV: levofloxacin; AMK: amikacin; ATM: aztreonam; IMP: imipenem; ETP: ertapenem; MEM: meropenem; CAZ-AVI: ceftazidime avibactam; TGC: tigecycline; PB: polymyxin B.

A total of 17 strains of CRE were detected, including 8 strains of *Klebsiella pneumoniae*, 7 strains *of Escherichia coli*, 1 strain of *Enterobacter cloacae*, and 1 strain of *Enterobacteriaceae* (Figure 2). Seven strains of *Klebsiella pneumoniae*, three strains of Escherichia coli, and one strain of *Enterobacter coli* were collected for antimicrobial susceptibility testing. All 11 CRE colonization strains were sensitive to tigecycline and polymyxin. *Klebsiella pneumoniae* producing the *Klebsiella pneumoniae* carbapenemases (KPC) were sensitive to ceftazidime avibactam, and *Escherichia coli* producing the new delhi metallo-β-lactamase (NDM) were all resistant to it. All strains were resistant to tazobactam, sulbactam, and cephalosporins. Some strains were sensitive to quinolones and aminoglycosides (Figure 3).

### 2.5. Molecular Characteristics and Phylogenetic Analysis

**Figure 4:**
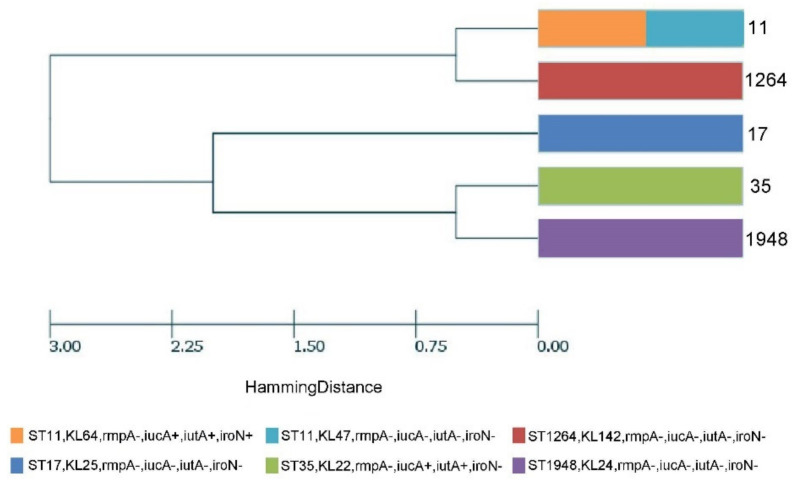
Multilocus sequence typing (MLST), capsular serotyping, virulence factors, and phylogenetic analysis of the six colonization strains of *Klebsiella pneumoniae* isolated from the ESLD patients. The numbers listed in right are MLST type.

Of the 11 CRE strains collected, 4 strains of *Klebsiella pneumoniae* and 2 of *Escherichia coli* produced the NDM enzyme. Two strains of *Klebsiella pneumoniae* produced KPC. One strain of Klebsiella pneumoniae, one of *Escherichia coli*, and one of *Enterobacter cloacae* did not produce carbapenemases (Figure 2). Of the seven CRKP strains, multilocus sequence type (MLST) included ST17, ST 1948, ST1264, ST11, and ST35, and one strain belonged to the other clone, which cannot be typed at present (Figure 4). The phylogenetic tree, based on MLSTs, showed that the strains were not due to a clone outbreak. The strains of ST 11 and 1264, as well as strains 35 and 1948, were more closely related to each other. The capsular serotypes were KL25, KL24, KL142, KL64, KL47, and KL22, respectively. The virulence factors, iucA, iutA, and iroN were detected in one strain, while iucA and iutA were detected in another. No virulence factors were detected in the other CRKP strains. 

## 3. Discussion

Many previous studies on CRE colonization have focused on hematologic diseases and ICU populations, and the results have varied widely. For example, CRE colonization rates were reported as 1.5–75.5% in hematological malignancy patients [17,20]. CRKP is the most common CRE species [21]. It was reported that the CRKP carriage rate was 7% in ICUs in Israel and 28% in China [22,23]. To the best of our knowledge, research investigating CRE colonization in ESLD patients is mainly conducted in Germany [19,24]. Our study showed that the CRE colonization rate in ESLD patients was 5.5%, which was higher than the 2.8% in liver transplant candidates in Germany [24]. CRE infections have great regional differences [25]. The detection rate of *Klebsiella pneumoniae* in our study was similar to that in Germany, but the detection rate of *Escherichia coli* was significantly different [24]. 

Our data indicated that the length of hospital stay, invasive procedures, and artificial liver support were risk factors for CRE colonization, and artificial liver support was an independent risk factor. There is a consensus that hospital stay and invasive procedures are risk factors for CRE infection [26]. However, the relationship between an artificial liver and CRE acquisition has not been studied extensively. Dialysis, also as a blood purification treatment, is considered a risk factor for CRE carriage by the European Center for Disease Prevention and Control [27]. Antibiotic exposure and endogenous evolution are the main mechanisms of CRE colonization in the dialysis population [15]. In our study, 53 (17.04%) patients had artificial liver support. Compared with patients without an artificial liver, antibiotic exposure in the artificial liver group was significantly increased (*p* = 0.003, data not shown), suggesting that antibiotic exposure may contribute to CRE colonization. In addition, artificial liver support would remove some immune molecules of the patients and usually be performed with catheterization in a specialized treatment area, implying the possibility of environmental transmission [28,29,30]. Whether these factors contribute to CRE colonization requires further prospective cohort studies. 

Our data suggest that the etiology of liver disease, MELD score, and Child–Pugh score were not significantly different between the colonization and non-colonization groups. Logistic regression analysis also indicated that they were not risk factors for colonization. These results suggest that CRE colonization in ESLD may be independent of etiology and is associated with a common pathophysiology, that is, impaired immunity, gut microbiome dysfunction, and barrier impairment [1,2]. A cohort study of US veterans suggested that proton pump inhibitors (PPIs) are risk factors for CRE [31]. PPIs are commonly used for ESLD. Whether this will significantly increase the risk of CRE colonization in the ESLD population deserves further investigation.

In addition to the high rate of CRE colonization in ESLD patients and potential risk factors, we found an increased rate of CRE infection and a worse prognosis in the CRE colonization group, which was consistent with the findings of Ferstl et al. [19]. One recent study revealed that rectal colonization by NDM-KP was more prone to bloodstream infection than KPC-KP, and all the bloodstream infection strains belonged to ST147 [32]. Unfortunately, our sample size was too small to conduct such analysis. Only two patients had CRE infection, *Escherichia coli* pneumonia and *Klebsiella pneumoniae* wound infection, both of which were KPC-producing, and no ST147 strain was found. Further attention should be paid to whether some intestinal colonizers would lead to specific infections in the ESLD population.

The acquisition of CRE usually involves two methods: self-evolution and exogenous transmission. The CRE colonization patients in our study had relatively scattered hospital stays, and only two patients had an intersection of hospital stays. However, they belonged to different clones, ST17 and ST 1948, respectively. Hence, it is unlikely that CRE colonization was transmitted horizontally from our hospital. As a large liver disease center, we treat patients from all over southern China. Of the people we included, 16 (94.12%) of CRE colonization patients were admitted from a local hospital; thus, whether these strains were exogenously acquired remains uncertain.

In contrast to the European Society of Clinical Microbiology and Infectious Diseases and Infectious Disease Society of America 2022 guidelines [33,34], combination therapy with polymyxin or tigecycline is still recommended in China. Among the 11 strains analyzed, 6 (54.5%) strains produced the NDM enzyme and were resistant to ceftazidime-avibactam but sensitive to polymyxin and tigecycline. Treatment of these strains with ceftazidime–avibactam alone would result in treatment failure. Our results suggest that polymyxin and tigecycline can be used as alternative agents. All KPC-producing strains were sensitive to ceftazidime–avibactam, whereas NDM strains were resistant. Our study highlights the need to detect carbapenemase genes to guide CRE antimicrobial treatment.

This study had some limitations. First, we defined CRE colonization based on a single rectal screening. The colonization detection rate may increase with an increase in screening frequency [18]. Although we used the broth enrichment method before inoculation to maximize the positivity rate, the missed detection rate is still inevitable. Second, as a retrospective observational study, we failed to preserve all strains for further analysis of molecular characteristics. Our conclusion needs to be confirmed in future prospective cohort studies with larger sample sizes. 

## 4. Materials and Methods

### 4.1. Patients

This was a retrospective, observational study. A total of 311 hospitalized patients were enrolled for CRE colonization screening from October 2020 to January 2022 in the department of infectious diseases of The Third Affiliated Hospital of Sun Yat-sen University. Based on expert consensus on the diagnosis and treatment of ESLD complicated infection (2021 version) [35], patients who met one of the four criteria were included in this study: (1) acute-on-chronic liver failure, that is, serum total bilirubin greater than 10 times the upper limit of the normal range and plasma prothrombin activity ≤ 40% or international standardized ratio more than 1.5, or hepatic encephalopathy or hepatorenal syndrome on the basis of chronic liver disease; (2) acute decompensation of liver cirrhosis, that is, cirrhosis confirmed by ultrasound or computed tomography (CT) or magnetic resonance (MR) with acute liver dysfunction, including elevated serum alanine aminotransferase and total bilirubin, or sudden onset of ascites or peritonitis; (3) chronic liver failure, that is, cirrhosis with slowly progressive liver dysfunction and plasma prothrombin activity ≤ 40%, or international standardized ratio more than 1.5, or refractory ascites or portal hypertension or hepatic encephalopathy; and (4) hepatocellular carcinoma, including a nodule ≤ 2cm on the basis of chronic liver disease with at least two imaging manifestations (ultrasound, CT, or MR), a nodule > 2 cm with at least one imaging manifestations (ultrasound, CT, or MR) with elevated alpha-fetoprotein (excluding other causes), or a pathological biopsy of the liver indicating liver malignancy. The exclusion criteria were as follows: (1) pregnancy; (2) initial negative CRE colonization screening result with follow-up dynamic screening result as positive. Clinical records were extracted from the electronic database of our institution. Colonization and infection were defined as previously described [24]. Deteriorated discharge, liver transplantation, or death were defined as unfavorable prognosis, while improved discharge was defined as good prognosis. The study was approved by the Ethics Committee of The Third Affiliated Hospital of Sun Yat-sen University ([2021]02-309-01) and strictly adhered to the Declaration of Helsinki. Informed consent was waived by the Ethics Committee. The study was registered in the Chinese Clinical Trial Registry (ChiCTR2100047284).

### 4.2. Screening Procedure and Antimicrobial Susceptibility Test

Two to six grams of stool was inoculated in broth (Dijing, Guangzhou, China) for 6 h, and then 10 ul of stool was inoculated into the MacConkey plate (Dijing, Guangzhou, China) between the first and second zones with meropenem paper (10 ug) for 16–24 h. Suspicious colonies with inhibition zones less than 22 mm were identified by matrix-assisted laser desorption/ionization time-of-flight mass spectrometry (MALDI-TOF MS) (Bruker, Germany). If necessary, the colonies were identified after separation. Antimicrobial susceptibility was examined using the broth microdilution method. The antimicrobial results were interpreted according to the guidelines of the Clinical and Laboratory Standards Institute (CLSI, 2021), except for tigecycline, which was interpreted according to the FDA criteria. *Escherichia coli* ATCC 25922 and *Pseudomonas aeruginosa* ATCC 27853 were used for quality control. 

### 4.3. Molecular Characteristics and Phylogenetic Analysis

Carbapenem-related resistance genes (IMP, SPM, VIM, BIC, NDM, KPC, AIM, GIM, SIM, DIM, and OXA-48), MLST-related genes (mdh, infB, pgi, gapA, rpoB, phoE, tonB), virulence factors (rmpA, iucA, iutA, and iroN), and capsular serotype-related genes (wzi) were detected by polymerase chain reaction (PCR). The PCR products were sequenced in BGI TECH SOLUTIONS (Liuhe, Beijing, China). The sequencing results were blasted at https://blast.ncbi.nlm.nih.gov/Blast.cgi, accessed on 1 September 2022; and https://pubmlst.org/bigsdb?db=pubmlst_mlst_seqdef&page=sequenceQuery, accessed on 15 September 2022. The primers used are listed in Table S1 [36]. The phylogenetic tree was analyzed using PHYLOVIZ software based on the MLST sequence.

### 4.4. Statistical Analysis

SPSS version 26.0 was used for statistical analyses. Data are expressed as means ± standard deviation (SD) or frequencies and percentages according to variable properties. A Student’s t-test or chi-squared test was used to analyze the difference between CRE colonization and non-CRE colonization groups. Univariate and multivariate logistic regression methods were used to identify risk factors for CRE colonization, while the univariate method was used to identify risk factors for CRE infection and the prognosis of patients with ESLD; *p* values < 0.05 were considered significant.

## 5. Conclusions

There was a high colonization rate in ESLD patients, and the colonization strains were highly diverse. CRE colonization deserves attention in these patients, especially when treated with artificial liver support. 

## Figures and Tables

**Table 1:** Demographic and clinical characteristics of CRE colonization and non-CRE colonization patients with ESLD.

Variables	Total	Non-CRE Colonization	CRE Colonization	*p*-Value
Patients	311	294	17	NA
Age (years)	51.08 ± 12.10	51.27 ± 12.12	47.82 ± 11.56	0.255
Male	255 (81.99%)	240 (81.63%)	15 (88.24%)	0.491
Hepatitis B or C	256 (82.32%)	242 (82.31%)	14 (82.35%)	0.997
Cirrhosis	205 (65.92%)	196 (66.67%)	9 (52.94%)	0.246
Charlson comorbidity index	3.00 (3.00–4.00)	3.00 (3.00–5.00)	3.00 (3.00–3.00)	0.096
Diabetes	45 (14.47%)	45 (15.30%)	0	0.146
Tumor	61 (19.61%)	60 (20.41%)	1 (5.88%)	0.143
Admitted from hospital	295 (94.86)	279 (94.90)	16 (94.12)	0.887
Length of hospital stay	7.00 (2.00–13.00)	7.00 (2.00–13.00)	10 (2.50–30.00)	0.305
WBC (*e9/L)	5.41 (3.61–8.17)	5.42 (3.60–8.21)	5.30 (3.75–8.63)	0.667
Hb (g/L)	98.00 (81.00–115.00)	98.00 (81.75–115.00)	101.00 (80.50–109.50)	0.795
PLT (*e9/L)	85.00 (56.00–131.00)	85 (56.00–131.00)	81 (50.50–118.00)	0.496
ALT (U/L)	41.00 (23.00–95.00)	41.00 (22.75–95.00)	35.00 (27.00–75.00)	0.886
AST (U/L)	68.00(38.00–114.00)	67.5 (37.00–114.00)	87 (53.00–119.50)	0.364
ALB (g/L)	34.49 ± 5.29	34.56 ± 5.28	33.25 ± 5.37	0.320
TB (umol/L)	154.40 (42.40–331.00)	150.85 (39.53–327.85)	198.00 (80.50–525.00)	0.146
CHE (U/L)	2968.00 (1928.00–4191.00)	2974.25 (1927.25–4140.25)	2571 (1921.50–4462.00)	0.907
HDL (mmol/L)	0.24 (0.12–0.55)	0.24 (0.13–0.59)	0.14 (0.12–0.26)	0.055
LDL (mmol/L)	1.47 (0.99–2.05)	1.47 (1.00–2.05)	1.1 (0.80–1.83)	0.141
Cr (umol/L)	67.00 (55.00–85.00)	67.00 (55.00–85.25)	60.00 (48.75–88.00)	0.445
INR	1.67 (1.32–2.22)	1.67 (1.32–2.21)	2.10 (1.35–2.40)	0.432
MELD score	22.00 (13.45–32.31)	21.53 (13.30–31.97)	27.37 (14.22–39.51)	0.240
Child–Pugh score	9.00 (8.00–11.00)	9.00 (8.00–11.00)	10.00 (9.00–11.50)	0.182
ICU admission	10 (3.22%)	10 (3.40%)	0	1.000
Gastrointestinal bleeding	27 (8.68%)	26 (8.84%)	1 (5.88%)	0.673
Blood transfusion	272 (87.46%)	256 (87.07%)	16 (94.12%)	0.394
Parenteral nutrition	20 (6.43%)	19 (6.46%)	1 (5.88%)	0.924
Immunosuppressive or antitumor therapy	44 (14.15%)	40 (13.61%)	4 (23.53%)	0.254
Invasive procedures	179 (57.56%)	165 (56.12%)	14 (82.35%)	0.033
Central venous catheterization	133 (42.77%)	123 (41.84%)	10 (58.82%)	0.169
Artificial liver support	53 (17.04%)	45 (15.31%)	8 (47.06%)	0.001
Antibiotic therapy	238 (76.53%)	221 (75.17%)	17 (100.00%)	0.019
Carbapenems	85 (27.33%)	79 (26.87%)	6 (35.29%)	0.449
Penicillins	4 (1.29%)	4 (1.36%)	0	1.000
Cephalosporins	44 (14.15%)	39 (13.27%)	5 (29.41%)	0.063
Comprised β-lactamases antibiotics	184 (59.16%)	172 (58.50%)	12 (70.59%)	0.324
Quinolones	26 (8.36%)	26 (8.84%)	0	0.378
CRE infection	2 (0.64%)	1 (0.34%)	1 (5.88%)	0.005
Unfavorable outcome	79 (25.40%)	71 (24.15%)	8 (47.06%)	0.035

NA: not applicable; WBC: white blood cell; Hb: hemoglobin; PLT: platelet; ALT: alanine aminotransferase; AST: aspartate aminotransferase; ALB: albumin; TB: total bilirubin; CHE: cholinesterase; HDL: high density lipoprotein cholesterol; LDL: low density lipoprotein cholesterol; Cr: creatinine; INR: international normalized ratio of prothrombin time; MELD: the Model for End-Stage Liver Disease.

**Table 2:** Univariate and multivariate analysis of risk factors for CRE colonization in patients with ESLD.

Variables	Univariate Analysis			Multivariate Analysis		
	*p*-Value	Hazard Ratio	95% CI	*p*-Value	Hazard Ratio	95% CI
Age (years)	0.256	0.977	0.938–1.017			
Gender	0.496	1.687	0.375–7.598			
Hepatitis B or C	0.997	1.003	0.278–3.615			
Cirrhosis	0.251	0.563	0.211–1.503			
Charlson comorbidity index	0.380	0.825	0.538–1.267			
Admitted from hospital	0.887	0.860	0.107–6.927			
Length of hospital stay	0.032	1.026	1.002–1.051	0.200	1.018	0.991–1.045
WBC (*e9/L)	0.603	1.030	0.921–1.153			
ALT(U/L)	0.394	0.997	0.990–1.004			
TB (umol/L)	0.105	1.002	1.000–1.004			
Cr(umol/L)	0.524	0.995	0.979–1.011			
INR	0.855	0.986	0.849–1.145			
MELD score	0.233	1.025	0.984–1.067			
Child–Pugh score	0.125	1.236	0.943–1.621			
ICU admission	0.999	0	NA			
Gastrointestinal bleeding	0.676	0.644	0.082–5.055			
Blood transfusion	0.408	2.375	0.306–18.427			
Parenteral nutrition	0.924	0.905	0.114–7.191			
Immunosuppressive or antitumor therapy	0.262	1.954	0.607–6.290			
Invasive procedures	0.045	3.648	1.027–12.966			
Central venous catheterization	0.176	1.986	0.736–5.363			
Artificial liver support	0.002	4.919	1.802–13.422	0.009	4.071	1.427–11.618
Surgery	0.254	3.612	0.398–32.775			
Antibiotic therapy	0.997	124267258	NA			
Carbapenems	0.451	1.484	0.531–4.148			
Penicillins	0.999	0	NA			
Cephalosporins	0.073	2.724	0.910–8.155			
Comprised β-lactamases antibiotics	0.329	1.702	0.585–4.957			
Quinolones	0.998	0	NA			

WBC: white blood cell; ALT: alanine aminotransferase; TB: total bilirubin; Cr: creatinine; INR: international normalized ratio of prothrombin time; MELD: the Model for End-Stage Liver Disease.NA, not applicable.

## Data Availability

The data in the study are available from the corresponding author on reasonable request.

## Supplementary Materials

The following supporting information can be downloaded at https://www.mdpi.com/article/10.3390/antibiotics11111667/s1, Table S1: Primers of carbapenem resistance, multilocus sequence type, capsular serotype related genes and virulence factors in this study.
