# Supplementary material for: Colonization with Carbapenem-Resistant Enterobacteriaceae Contributes to Unfavorable Outcomes in End-Stage Liver Disease Patients
Source: Antibiotics (Basel). 2022 Nov 20;11(11):1667. doi: 10.3390/antibiotics11111667 (PMC9686982; doi:10.3390/antibiotics11111667)
Supplement: Supplementary file 1 [file antibiotics-11-01667-s001.zip › antibiotics-1982147-supplementary.pdf]

Table S1. Primers of carbapenem resistance, multilocus sequence type, capsular serotype related genes and virulence factors in this study.

Primers	Sequence (5′>3′)	Fragment size (bp)
IMP-F	GGAATAGAGTGGCTTAAYTCTC	232
IMP-R	GGTTTAAYAAAACAACCACC	
SPM-F	AAAATCTGGGTACGCAAACG	271
SPM-R	ACATTATCCGCTGGAACAGG	
VIM-F	GATGGTGTTTGGTCGCATA	390
VIM-R	CGAATGCGCAGCACCAG	
BIC-F	TATGCAGCTCCTTTAAGGGC	537
BIC-R	TCATTGGCGGTGCCGTACAC	
NDM-F	GGTTTGGCGATCTGGTTTTC	621
NDM-R	CGGAATGGCTCATCACGATC	
KPC-F	ATGTCACTGTATCGCCGT	846
KPC-R	GGTGGTGGGCCAATAGAT	
AIM-F	CTGAAGGTGTACGGAAACAC	322
AIM-R	GTTCGGCCACCTCGAATTG	
GIM-F	TCGACACACCTTGGTCTGAA	477
GIM-R	AACTTCCAACTTTGCCATGC	
SIM-F	TACAAGGGATTCGGCATCG	570
SIM-R	TAATGGCCTGTTCCCATGTG	
DIM-F	GCTTGTCTTCGCTTGCTAACG	699
DIM-R	CGTTCGGCTGGATTGATTTG	
OXA-48-F	GCGTGGTTAAGGATGAACAC	438
OXA-48-R	CATCAAGTTCAACCCAACCG	
mdh-F	CCCAACTCGCTTCAGGTTCAG	800
mdh-R	CCGTTTTTCCCCAGCAGCAG	
infB-F	CTCGCTGCTGGACTATATTCG	506
infB-R	CGCTTTCAGCTCAAGAACTTC	
pgi-F	GAGAAAAACCTGCCTGTACTGCTGGC	610
pgi-R	CGCGCCACGCTTTATAGCGGTTAAT	
gapA-F	TGAAATATGACTCCACTCACGG	706
gapA-R	CTTCAGAAGCGGCTTTGATGGCTT	
rpoB-F	GGCGAAATGGCWGAGAACCA	1119
rpoB-R	GAGTCTTCGAAGTTGTAACC	
phoE-F	ACCTACCGCAACACCGACTTCTTCGG	646
phoE-R	TGATCAGAACTGGTAGGTGAT	
tonB-F	CTTTATACCTCGGTACATCAGGTT	583
tonB-R	ATTCGCCGGCTGRGCRGAGAG	
wzi-F	GTGCCGCGAGCGCTTTCTATCTTGGTATTCC	580
wzi-R	GAGAGCCACTGGTTCCAGAA[C or T]TT[C or G]ACCGC	
rmpA-F	ACGACTTTCAAGAGAAATGA	516

rmpA-R	CATAGATGTCATAATCACAC	
iutA-F	ACCTGGGTTATCGAAAACGC	956
iutA-R	GATGTCATAGCCTGATTGC	
iucA-F	ATAAGGCAGGCAATCCAG	940
iucA-R	CGCTTCACTTCTTTCACTGACAGG	
iroN-F	AAGTCAAAGCAGGGGTTGCCCG	655
iroN-R	GACGCCGACATTAAGACGCAG	
